# Energy Density and Nutrient Contents of Selective Chinese New Year Snacks

**DOI:** 10.3390/foods9081137

**Published:** 2020-08-18

**Authors:** Michelle Ting Yun Yeo, Penny Liu Qing Yeo, Xinyan Bi, Christiani Jeyakumar Henry

**Affiliations:** 1 Clinical Nutrition Research Centre (CNRC), Singapore Institute of Food and Biotechnology Innovation (SIFBI), Agency for Science, Technology and Research (A*STAR), 14 Medical Drive, Singapore 117599, Singapore; michelle_yeo@sifbi.a-star.edu.sg (M.T.Y.Y.); penny_yeo@sifbi.a-star.edu.sg (P.L.Q.Y.); Bi_xinyan@sifbi.a-star.edu.sg (X.B.); 2 Department of Biochemistry, Yong Loo Lin School of Medicine, National University of Singapore, Singapore 117599, Singapore

**Keywords:** energy density, fatty acid content, elemental composition, Chinese New Year Snacks, Calorie Answer, ICP-MS, GC-MS

## Abstract

Background: In this study, the energy density and nutrient contents of thirty Chinese New Year (CNY) snacks commonly consumed in the Asian region during the CNY festive season were measured. Methods: Calorie Answer™, Gas Chromatography-Mass Spectrometry (GC-MS) and Inductively coupled plasma-mass spectrometry (ICP-MS) were the main methods applied in this study. Results: All snacks showed high energy density (kJ/100 g) and the nutrient content, including macro-and micronutrients of these snacks were remarkably different. The most abundant minerals in these snacks include sodium, magnesium, potassium, and calcium. Palmitic (C16:0), oleic (C18:1) and linoleic (C18:2) acids were the main fatty acids and *trans*-fat was only identified in Butter cookies. Conclusions: This study provides a large database on macro- and micronutrient contents in CNY snacks consumed in the Asian region that has not been previously reported. Our results indicate that the snacks were generally energy-dense and nutrient-poor. This study provides necessary information to enable the reformulation of snacks with lower saturated fat and sodium content. It is also a source of information for consumers to select healthier snacks.

## 1. Introduction

Chinese New Year (CNY), also known as Lunar New Year or Spring festival, is a traditional festival that is celebrated for fifteen days. It is an important period of time when families gather and enjoy seasonal treats and snacks. Besides CNY, snacks are also consumed during other festivals. For example, during Ramandan, Muslims will consume snacks such as cookies at the end of the day to celebrate. For the Indian hindus, snacks are commonly consumed during Deepavali (festival of lights) and Pongal (harvest festival).

Worldwide, snacks are increasingly being consumed in times besides during festive seasons. In Singapore, 27.1% of adult Singapore residents consumed sweet desserts and snacks three times or more per week [1]. In India, total fat intake increased by 47.9% and 31.9% in rural and urban populations respectively over the past three decades [2]. One of the reasons for this increase is due to the increased consumption of convenience foods. In China, there is a similar trend, where consumers are including more processed foods in their diets [3]. Since processed/convenience foods such as snacks are increasingly consumed, it is of utmost importance to investigate the macro- and micro-nutrient content of these items.

Recently, the snack industry has been enriching snacks with micronutrients, i.e., mineral-enriched snacks [4]. Monitoring the mineral content of snacks is a matter of public concern. Diet-related chronic diseases, e.g., high blood pressure, are affecting many people in Asia due to the overconsumption of sodium [5]. Other than sodium, the mineral contents of snacks are of interest since they serve as cofactors for many physiologic and metabolic functions [6]. However, there is limited information on the mineral content of these snacks because usually the micronutrient content is not reflected on the food labels, unless specific health claims are made [7].

The evaluation of the nutrient and energy intake of the population and individuals is a task that demands accurate information about the nutrient contents of foods. Snacks are habitually consumed by a majority of the population [8]. However, most nutrient data are largely assembled by a combination of online sources and Atwater Conversion Factors. There is also limited research conducted on the energy density and nutrient content of snacks. Hence, the objective of this study was to determine the energy density (kJ/100 g), macronutrients content (fat, protein, and carbohydrates), mineral content (Na23, Mg24, Al27, K39, Ca40, Mn55, Fe56, Cu63, Zn66) and fatty acid profile of thirty CNY snacks commonly consumed in Asia during the CNY period.

## 2. Materials and Methods

### 2.1. Sample and Sample Preparation

Thirty CNY snacks commonly consumed during the CNY period were purchased from a local supermarket (NTUC FairPrice, Singapore), which is one of the common supermarket brands in Singapore. All of the samples were homogenized by a miller (Knife Mill, Grindomix GM 200, Retsch, Haan, Germany) to obtain a smooth, consistent texture. Samples were stored at −20 °C before analysis.

### 2.2. Reagents for Inductively Coupled Plasma-Mass Spectrometry (ICP-MS)

High purity HNO_3_ (≥70% *w*/*w*, TRACEMETAL™ Grade, Fisher Scientific, Waltham, MA, USA) and HCl (≥38% *w*/*w* TRACEMETAL™ Grade, Fisher Scientific, Waltham, MA, USA) were used as received. The internal standard solution was prepared by appropriate dilution of the Internal Standard Mix solution (Agilent Technologies, Santa Clara, CA, USA). Calibration standards were prepared from 10 µg/mL and 100 µg/mL custom mix multi-element ICP-MS standard (HPS, North Charleston, SC, USA), and from the 1000 µg/mL mineral element ICP-MS standard solution (ICP-AM-17 Mineral Calibration Standard, HPS, North Charleston, SC, USA). All solutions were prepared by using ultrapure water (>18.2 MΩ·cm at 25 °C) from a Mili-Q IQ-7000 (Merck KGaA, Darmstadt, Germany) water purification system. All the glassware and plastic materials were decontaminated with a 10% (*v*/*v*) HNO3 solution for 48 h and rinsed with ultrapure water prior to use.

### 2.3. Reagents and Standard Solutions for Gas Chromatography-Mass Spectrometry (GC-MS)

The internal standard solution was prepared by dissolving 200 mg of methyl undecanoate (C11:0 FAME, Sigma Aldrich, Singapore) in 1 mL of n-hexane (Merck, KGaA, Darmstadt, Germany). The fatty acid methyl esters (FAMEs) standard solution was prepared by diluting the stock standard (GLC 36, Nu-Chek-Prep, Elysian, MN, USA) in n-hexane (Merck, KGaA, Darmstadt, Germany). Lipid extraction was performed using petroleum spirit (40 °C–60 °C, Avantor VWR, Radnor, PA, USA). For the determination of fatty acids profile, sodium hydroxide (Merck, KGaA, Darmstadt, Germany), boron trifluoride (BF3) (Merck, KGaA, Darmstadt, Germany), methanol (Merck, KGaA, Darmstadt, Germany), n-hexane (Merck, KGaA, Darmstadt, Germany) and ultrapure water (>18.2 MΩ·cm at 25 °C) from a Mili-Q IQ-7000 (Merck KGaA, Darmstadt, Germany) water purification system was used.

### 2.4. Microwave-Assisted Acid Digestion for ICP-MS Pretreatment

For the digestion of the snack samples, a CEM MARS6 Microwave Digestor System coupled with iPrep 12 vessels rotor (CEM, Matthews, NC, USA) was used. Of these twelve vessels, ten were samples, one was blank, and one was the 0.5 ppm spike sample. This configuration was kept unchanged in each digestion procedure. The sample (0.25 g) was weighed into the Teflon vessel, and 10 mL of HNO3 was then added into each vessel. The mixture was heated by ramping up to 210 °C in 15 min and holding it for an additional 15 min. After cooling, sample solutions were diluted to 50 mL in decontaminated 50 mL skirted centrifuge tubes with 5% HNO3 and 0.5% HCl. All samples were digested in duplicates.

### 2.5. Lipid Extraction

Lipids were extracted with petroleum spirit using the Soxtec 2055 (FOSS, Hillerød, Denmark) in duplicates. The lipid extraction method was adapted from Shin et al. [9]. The sample (2.5 g) was weighted into each cellulose thimble and placed into the oven at 103 °C for 2 h. Defatted cotton was added into the thimbles before they were loaded onto the Soxtec, which was set at 135 °C. There were four basic steps in the extraction of lipids using the Soxtec. Firstly, the thimbles were lowered into boiling petroleum spirit. Next, the thimbles were lifted up so that the condensed petroleum spirit could rinse the sample. The petroleum spirit was then recovered and drained off. Lastly, the extraction cups were lifted to evaporate the rest of the petroleum spirit in the extraction cups using radiant heat. At the end of the Soxtec extraction, the extraction cups were placed into the oven at 103 °C for 30 min and then placed into the desiccator. The extracted lipids were stored at −20 °C until analysis.

### 2.6. Determination of Fatty Acid Profile

The fatty acid profile was determined based on an adaptation of an application note by Agilent [10]. The extracted lipids were diluted in a 1:1 ratio with n-hexane. 10 µL of each 1:1 mixture was placed into a GC vial and 3.3 µL of the internal standard solution was added. The vials were then loaded onto the autosampler of the GC-MS and the fatty acids were derivatised using the Agilent Sample Prep WorkBench. The steps were as follows: Firstly, 120 µL of 2N NaOH in methanol was added to the vial and then subjected to mixing for 20 s at 1500 rpm. The vial was heated at 70 °C for 5 min and 240 µL of 12.5% BF3 in methanol was added after the vial was cooled for 5 min. The vial was then subjected to mixing for 20 s at 1500 rpm and subsequently heated at 70 °C for 5 min. The vial was cooled for 5 min before 300 µL of water and 300 µL of hexane was added. The vial was mixed for 20 s at 1500 rpm and the resulting mixture was left to stand for 2 min before 1 µL of the upper layer was injected into the GC-MS.

### 2.7. GC-MS Analysis

Analysis of samples were carried out on a 7890 B GC system coupled to a 5977 B MS detector with MSD Chemstation software. The GC-MS parameters were adapted from Agilent [10]. Separation of the different FAMEs was performed on a HP-88 column (60 m × 0.25 mm, 0.20 µm, Agilent Technologies, Santa Clara, CA, USA). A split injector at 250 °C and a spilt ratio of 50:1 was used. The oven temperature program was as follows: isothermal at 140 °C for 5 min and increased to 240 °C at a rate of 4 °C/min and maintained for 0 min. Helium was used as the carrier gas under a constant flow mode at 1 mL/min. The MS detector was maintained at 280 °C and a scan acquisition mode of 40 to 500 AMU was used. The FAMEs in the samples were identified by comparing their retention times and mass spectrum with the calibration standards. In this study, peaks that cannot be identified were not considered in the calculation of the percentage of fatty acids.

### 2.8. ICP-MS Analysis

Inductively coupled plasma-mass spectrometry (ICP-MS) analyses were performed using the 7900 ICP-MS instrument (Agilent Technologies, Hachioji, Japan) equipped with Ultra High Matrix Introduction (UHMI) option. The standard sample introduction system was used throughout, consisting of a MicroMist nebulizer, quartz spray chamber and quartz torch with 2.5 mm internal diameter injector. Platinum tipped interface cones were used. High purity (99.9997%) argon (Air Liquide, Singapore) was used as the plasma source. The ICP-MS instrument operational parameters were as follow: RF power—1550 W; Sampling depth—10 mm, Carrier gas—0.90 L/min. An Agilent SP4 auto-sampler was used to deliver the samples, which were held in 50/15 mL tubes. Sc45, Ge72, Y89, In115, Bi209 were used as internal standards. The instrument was tuned for maximum signal sensitivity and stability, using the Tuning Solution (1 µg/L Ce, Co, Li, Mg, Tl, Y in 2% HNO_3_) for the ICP-MS (Agilent Technologies, Hachioji, Japan).

### 2.9. Method Validation and Statistical Analysis for Mineral Content Results

The ICP-MS analysis method used was adapted from the application note from Agilent [11]. The method validation was performed by evaluating the accuracy, precision, limit of detection (LOD), limit of quantitation (LOQ) and the correlation coefficients. Two of the thirty snacks was used to verify the accuracy for the determination of Na, Mg, Al, K, Ca, Mn, Fe, Cu, and Zn. Analyte addition and recovery experiments were performed. The average recoveries were presented in Tables S1–S3. The analyses for the determination of the total concentrations were performed in triplicates. All the results are expressed as the mean ± standard deviation.

For the Ca40 measurement, we used the atomic abundance of the Calcium element to convert the results of Ca44 to Ca40. The formula is as below:

$$\mathrm{Ca}40\;mineral\;concentration\;\left(ppm\right)\;=\;\frac{Ca^{44}\;mineral\;concentration\;\left(ppm\right)\times 96.94\%}{2.09\%}$$

### 2.10. NIR Spectroscopy and Analysis

Near-infrared (NIR) spectra of homogenized samples were obtained with Calorie Answer™ (CA-HM, JWP, Hirakawa, Aomori, Japan) over a wavelength range of 1100–2200 nm with a resolution of 7.5 nm and a data interval of 2 nm. The main components of the instrument include a halogen lamp as radiation source; acousto-optic tunable filter (ATOF) as a wavelength sensor, and light receiving sensors as light detectors. The reflectance mode was used for solid samples and the reference reflectance data was obtained with a calcium carbonate filled cell. Triplicates of each sample were scanned in cylindrical sample cells (internal diameter = 50 mm, depth 10 mm for solid samples). The Snacks and Confectionery settings were selected for all the samples. The inbuilt computer software (CA-HM Measurement Application Software, JWP, Hirakawa, Aomori, Japan) was set so that each triplicated portion was scanned 10 times, which were then averaged to give a mean spectrum to improve accuracy. The data was transformed into log 1/R and calorie density for each sample was then calculated in accordance with regression expressions pre-programmed in the software. The analysis time for each measurement (including time for calibration) was about 5 min. The procedures used were elaborated intensively by Lau. et al. [12].

### 2.11. Principal Component Analysis (PCA)

Principal Component Analysis (PCA) is a method of summarizing the data and it is used to identify and understand the underlying structure and pattern in the data. PCA reduces the number of variables in a dataset into smaller principal components. Since multiple variables were investigated in the study, PCA was used to analyse the relationship between the different variables. The first principal component explains much of the variance in the dataset followed by the next and so forth. The relationship between the observations and the principal components were visually represented by the scoreplot. Hence, observations which share similar characteristics will be visible through the score plot. Loadings plot visually represents the relationship between the variables and the principal components. This allows one to observe which variables are positively correlated with each other and which are inversely correlated. Furthermore the influence of the variables on the components can be observed from the distance between the variable and the origin (the center of the plot). PCA analysis and the plots in this study was done using “FactoMineR” package in R version 1.2.5033 (statistical software).

## 3. Results

This study revealed that snacks are generally energy-dense and nutrient-poor (Table 1). The photo images of the thirty CNY snacks can be found in the supporting information (Figure S1). The energy density of twenty-one CNY snacks were within the range of 2011 kJ/100 g to 2743 kJ/100 g. Among the thirty CNY snacks, Salted egg fish skin had the highest energy density (2743 kJ/100 g), while Cake bangkit had the lowest energy density (1587 kJ/100 g). Table 1 shows that the macronutrient compositions of the CNY snacks were remarkably different. The protein content ranged from 0.1 g/100 g to 42.6 g/100 g. The carbohydrate content ranged from 4.5 g/100 g to 85.3 g/100 g and the fat content ranged from 7.7 g/100 g to 51.9 g/100 g measured by Calorie Answer^TM^.

Table 1 shows the fat content measured by both Calorie Answer™ and Soxtec extraction was similar for all samples except for Arrowhead cracker and Peanut cookies. It was also observed that more than half of the samples had a fat content of more than 20 g/100 g.

**Figure 1:**
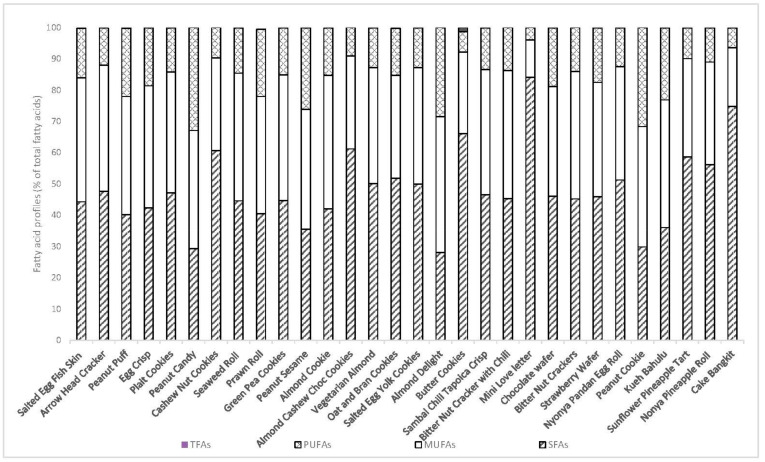
Fatty acid profiles (% of total fatty acids) of thirty CNY snacks.

Table 2 shows the average fatty acid composition of the thirty snacks. Fatty acids with a composition of less than 1% were not shown but they were included in the calculation of parameters such as total of saturated fatty acids (SFAs), total of monounsaturated fatty acids (MUFAs) and total of polyunsaturated fatty acids (PUFAs). Figure 1 shows the sum of SFAs, sum of MUFAs and sum of PUFAs. The fatty acid profiles of the thirty snacks were largely consistent and most of the snacks contained Myristic acid (C14:0), Palmitic acid (C16:0), Stearic acid (C18:0), Oleic acid (C18:1) and Linoleic acid (C18:2). Based on Figure 1, the amount of SFAs ranged from 28.03% in Almond delight to 84.09% in Mini love letter. The predominant type of fatty acid in the majority of snacks was SFAs except for Almond cookies, Kueh bahulu, Peanut sesame, Peanut cookies, Peanut candy and Almond delight. The predominant type of fatty acid in these six snacks was MUFAs.

C16:0 was the major saturated fatty acid found in all samples except for Mini love letter and Cake bangit where the major saturated fatty acid was C12:0. As for the MUFAs, the most abundant fatty acid was C18:1 and Almond delight contained the highest amount of C18:1 (42.24%) among the thirty snacks. The predominant fatty acid of PUFAs was C18:2 and Peanut candy contained the highest amount of C18:2 (32.77%). Twenty-two snacks contained C18:1 *trans* isomer but only Butter cookies contained more than 1% of C18:1 *trans* isomer (1.37%).

The mineral contents of the CNY snacks were shown in Table 3. The sodium content in the snacks ranged from 0.4 mg/100 g to 551.8 mg/100 g, with seventeen snacks having a sodium content more than 100 mg/100 g. Out of all thirty snacks, Salted egg fish skin had the highest amount of sodium (551.8 mg/100 g), while Bitter nut crackers had the lowest amount of sodium (0.4 mg/100 g). There were considerable variations in other mineral contents. For example, the magnesium content ranged from 8.2 to 83.8 mg/100 g, the potassium content ranged from 44.5 to 1300.6 mg/100 g, and the calcium content ranged from 45.1 to 734.8 mg/100 g (Table 3). In this study, we found that the snacks were high in macro elements, such as sodium, potassium, magnesium, and calcium, but low in the trace elements, such as copper, iron, manganese, and zinc. For example, the iron and zinc contents in Sunflower pineapple tart were 0.5 mg/100 g and 0.1 mg/100 g respectively. However, Prawn roll contained 734.8 mg/100 g of calcium and the consumption of 50 g of Prawn roll reached approximately 37% of the recommended daily level of calcium intake (Table 4).

**Figure 2:**
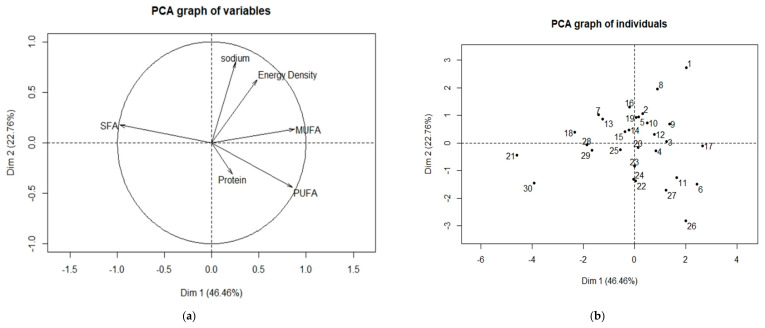
(**a**) PCA plot of different variables; (**b**) PCA plot of individual sample. (1) Salted Egg Fish Skin; (2) Arrowhead Cracker; (3) Peanut Puff; (4) Egg Crisp; (5) Plait Cookies; (6) Peanut Candy; (7) Cashew Nut Cookies; (8) Seaweed Roll; (9) Prawn Roll; (10) Green Pea Cookies; (11) Peanut Sesame; (12) Almond Cookies; (13) Almond Cashew Chocolate Cookies; (14) Vegetarian Almond Cookies; (15) Oat and Bran Cookies; (16) Salted Egg Yolk Cookies; (17) Almond Delight; (18) Butter Cookies; (19) Sambal Chili Tapioca Crisp; (20) Bitter Nut Cracker with Chili; (21) Mini Love Letter; (22) Chocolate Wafer; (23) Bitter Nut Crackers; (24) Strawberry Wafer; (25) Nyonya Pandan Egg Roll; (26) Peanut Cookies; (27) Kueh Bahulu; (28) Sunflower Pineapple Tart; (29) Nyonya Pineapple Roll; (30) Cake Bangkit.

Figure 2 shows the PCA plots using the following variables: energy density, MUFAs, PUFAs, SFAs, sodium and protein. Based on Figure 2a, it can be observed that the vectors representing energy density and sodium are close to each other, which seems to suggest that energy density and the level of sodium in an individual sample are positively correlated. This meant that an individual sample high in energy density would also be high in salt content. The vectors representing SFAs and PUFAs are opposite each other, which also suggests that the level of SFAs and PUFAs in an individual sample are negatively correlated. Figure 2b shows the distribution of the individual samples based on their relationship with the variables. Hence, by overlapping Figure 2a,b, the relationship of an individual sample and the variables could be shown. For example, by overlapping Figure 2a,b, it can be observed that Salted egg fish skin is associated with energy density and salt, which meant that it is of high energy density and contain high levels of salt. By referring back to our experimental results, it was observed that Salted egg fish skin had the highest energy density and sodium content out of the thirty snacks, which justified the PCA results. The PCA results also seems to suggest that Peanut cookie is the “healthier” snack choice, which coincided with our experimental results. Our findings showed that Peanut cookie contained high levels of PUFAs (of which most of it was made up of C18:2), low levels of sodium (3 mg/100 g), and comparably low levels of SFAs.

## 4. Discussion

To the best of our knowledge, this paper provides a detailed analysis of the energy and nutrient contents of selected widely consumed CNY snacks that have not been previously reported. Our study indicated that CNY snacks were energy-dense and contained higher energy density than staple meals (based on rice, noodles and potatoes). Quek et al. [13] reported the energy density of common staple meals in Asia such as grilled fish with rice (183 kcal/100 g or 765 kJ/100 g), egg prata (292 kcal/100 g or 1222 kJ/100 g) and prawn noodles 107 kcal/100 g or 448 kJ/100 g). These values were lower than the energy density of all thirty snacks investigated in this study. Hence, information from our study could be used to advise consumers to limit snacks consumption since some of the snacks listed in this study are commonly consumed other than during the CNY period.

Previous studies have shown that NIR analysis produced comparable results to Soxhlet extraction method for the determination of fat content in food such as potato chips and tuna fish [14,15]. In this study, the deviation between the fat content using NIR and Soxtec extraction method respectively for all samples were less than 10% except for Arrowhead cracker and Peanut cookies. Hence, our results suggest that NIR analysis can be used for the determination of fat content in snacks. This is because NIR analysis produces reproducible results and it is a much faster method compared to Soxtec extraction.

Snacks are often considered “unhealthy” foods due to their high energy density and poor nutrient quality. Besides macronutrients, minerals contents and fatty acid profile were investigated since they were known to play an important role in metabolism and in the maintenance of tissue function. The excessive sodium intake is of particular concern since sodium can lead to high blood pressure [16], which is an independent risk factor for cardiovascular disease. The recommendation for sodium intake is 2 g per day; therefore, salt intake should not exceed 5 g per day [17]. From our analysis, the consumption of 50 g of Salted egg fish skin reached approximately 11% of the recommended daily level of sodium intake. On the other hand, trace elements found in the samples such as iron and zinc were low. For example, the iron and zinc contents in Almond cookies were 0.6 mg/100 g and 0.2 mg/100 g respectively, which were below the recommended dietary intakes of iron (8 mg/day) and zinc (11 mg/day) for adults. These results, when combined, suggest that CNY snacks can be reformulated to reduce sodium (salt), but the amount of trace elements such as iron and zinc can be increased.

SFAs was the most abundant type of fatty acid in twenty-four out of the thirty snacks. The results were similar to another study where Dias, Passos, Tavares do Carmo, Lopes, & Mesquita [8] investigated sweet biscuits, salty biscuits and salty snacks and reported that SFAs was the most abundant type of fatty acids in the samples. In twenty-four snack samples where SFAs was the most abundant type of fatty acid, C16:0 was the predominant saturated fatty acid. C12:0, C14:0 and C18:0 were also found in the samples. High concentrations of these SFAs suggested that palm oil and palm kernel oil was used in the production of these cookies and snacks. High intake of saturated fat and low intake of polyunsaturated fat leads to an increase in serum cholesterol and this in turn leads to the development of atheromatous plague [18]. Food manufacturers could look at using alternatives like blending palm oil with liquid oils such as sunflower oil in the food products. Hinrichsen [19] mentioned that adding the resultant oil blend, made from fully hydrogenated oils and liquid oils, into the food product produces an end product that is acceptable by consumers.

In this study, the most abundant type of fatty acid in six of the snacks were MUFAs and the predominant MUFA in all samples was C18:1. Fernández [20] investigated the fatty acid profile of two hundred and eighty samples of commercial Spanish fast food and snack food and C18:1 was the main MUFA in all types of samples except for popcorn microwave samples.

Higher levels of MUFAs were detected in Almond cookies, Peanut sesame, Peanut cookies, Peanut candy and Almond delight because nuts like almonds and peanuts contain high levels of MUFAs in the form of oleic acid [21,22]. Higher levels of MUFAs were also detected in Kueh bahulu because eggs are a major component in kueh bahulu and eggs generally contain high amounts of oleic acid (42.6% to 45.2% of total fatty acids) [23]. Consumption of oleic acid seems to be associated with several health benefits. Lopes, Aro, Azevedo, Ramos, & Barros [24] reported that consumption of oleic acid is inversely associated with acute myocardial infarction. High oleic acid and olive oil consumption is also associated with a reduction in the risk of developing breast, colorectal and prostate cancer [25]. Hence, consumers should choose to consume snacks that contain higher amounts of MUFAs.

PUFAs were present in all of the samples and the predominant PUFA type was C18:2. The results in our study are in agreement with other studies [8,20]. C18:3 was also observed in the samples and based on Table 2, it ranged from 1.15% to 5.01%. Both linoleic acid and alpha linolenic acid are essential fatty acids that cannot be synthesised by the human body. This is because the human body is deficient in the desaturases needed to convert oleic acid to linoleic acid and linoleic acid to linolenic acid [26].

High intake of n-3 PUFAs will promote the production of anti-inflammatory eicosanoids while high intake of n-6 PUFAs promotes the production of pro-inflammatory eicosanoids [27]. An increase in the intake of n-3 PUFAs decreased the risk of breast cancer while an increase in the intake of n-6 PUFAs increased breast cancer risk [28]. Since the consumption of n-3 PUFAs and n-6 PUFAs have different effects on health but the formation of these fatty acids require the same enzymes, it is important to consume an appropriate ratio of n-3 PUFAs and n-6 PUFAs.

With reference to Table 2, the amount of C18:1 *trans* isomer in twenty-one snacks of this study was less than 1%. Previous studies have also reported similar values. Huang, Wang, Pace, & Oh [29] reported that the *trans* fatty acid level of several grocery foods and fast foods in an African-American community ranged from 0.51% to 1.82%. An average *trans* fatty acid value of 0.47 g/100 g of food was reported based on two hundred and sixty-eight commercialized food samples obtained from the Portugal community [30]. However, the studies also reported the presence of other *trans* fatty acids like C18:2 *trans* isomer, which were not observed in our study.

The main *trans* fatty acid derived from partial hydrogenation of vegetable oil is eladic acid (C18:1 *trans* 9) [31]. Hence, this means that the *trans* fatty acid found in the samples in our study were derived from the partial hydrogenation of vegetable oil. Brouwer, Wanders, & Katan [32] reported that *trans* fatty acids from both industrial and ruminant sources increased the LDL cholesterol: HDL cholesterol ratio. Intake of *trans* fatty acids is also associated with coronary heart disease mortality [33]. The low levels of *trans* fatty acids observed in our study might be the effect of ongoing initiatives and polices implemented by the Singapore government. In 2013, a regulation was amended to limit *trans* fat to no more than 2 g per 100 g product for fats and oils supplied to food manufacturers and food establishments and for fats and oils sold in the retail market [34].

## 5. Conclusions

In conclusion, our paper introduced a comprehensive analysis of the energy density and the nutrient contents of thirty commonly consumed CNY snacks in Asian region. The knowledge of the energy density and nutrient contents of the CNY snacks could help to produce good snacking strategies to decrease consumption of such snacks not only during the festive period but also in our daily lives. Our study also provides useful information for both the consumers to select healthier snacks and food manufacturers to improve on the energy density and nutrient contents of current snacks in the market. However, our study only managed to analyse thirty selected CNY snacks. Moving forward, we seek to investigate the energy density and nutrient contents of a larger number and wider variety of snacks.

## Figures and Tables

**Table 1:** Energy density and macronutrients contents of the commonly consumed CNY snacks. Values are expressed as mean ± SD.

Snack Item	Energy Density	Protein	Carbohydrate	Fat (Calorie Answer^TM^)	Fat (Soxtec)
	(kJ/100 g)	(g/100 g)	(g/100 g)		(g/100 g)
				(g/100 g)	
Salted Egg Fish Skin	2743 ± 20	42.6 ± 0.8	4.5 ± 0.4	51.9 ± 1.0	48.5 ± 1.0
Arrowhead Cracker	2582 ± 41	0.2 ± 0.0	43.5 ± 0.3	49.2 ± 1.2	36.8 ± 4.8
Peanut Puff	2466 ± 23	12.0 ± 2.5	48.3 ± 1.5	38.7 ± 1.1	40.1 ± 0.7
Egg Crisp	2364 ± 11	13.2 ± 0.6	52.0 ± 1.0	33.7 ± 0.5	29.2 ± 0.3
Peanut Candy	2332 ± 13	19.4 ± 0.7	47.3 ± 0.5	32.3 ± 0.6	32.0 ± 0.8
Cashew Nut Cookies	2318 ± 18	5.8 ± 0.7	61.6 ± 0.3	31.6 ± 0.9	30.9 ± 2.1
Seaweed Roll	2279 ± 9	10.1 ± 1.2	59.1 ± 1.7	29.7 ± 0.6	20.2 ± 1.3
Prawn Roll	2248 ± 2	21.6 ± 0.5	49.1 ± 0.5	28.3 ± 0.1	23.1 ± 0.6
Green Pea Cookies	2241 ± 42	4.3 ± 2.0	66.8 ± 0.4	28.0 ± 1.9	25.9 ± 1.0
Peanut Sesame	2220 ± 11	23.3 ± 0.1	48.7 ± 0.4	27.0 ± 0.5	34.7 ± 0.3
Almond Cookies	2216 ± 27	7.2 ± 2.2	65.1 ± 2.6	26.8 ± 1.3	28.6 ± 1.2
Almond Cashew Chocolate Cookies	2205 ± 4	24.2 ± 1.1	48.6 ± 0.9	26.2 ± 0.2	31.8 ± 0.2
Vegetarian Almond Cookies	2180 ± 4	9.0 ± 0.5	65.0 ± 0.7	25.0 ± 0.2	29.6 ± 0.3
Plait Cookies	2178 ± 9	3.8 ± 1.2	62.3 ± 1.3	33.0 ± 0.3	38.8 ± 0.4
Oat and Bran Cookies	2169 ± 10	12.7 ± 0.8	61.8 ± 1.2	24.4 ± 0.4	25.6 ± 1.0
Salted Egg Yolk Cookies	2156 ± 9	0.1 ± 0.1	75.1 ± 0.6	23.8 ± 0.5	23.9 ± 3.7
Almond Delight	2149 ± 3	14.8 ± 0.1	60.7 ± 0.2	23.5 ± 0.1	26.9 ± 0.9
Butter Cookies	2109 ± 0	13.9 ± 0.6	63.4 ± 0.6	21.6 ± 0.1	24.2 ± 0.3
Sambal Chili Tapioca Crisp	2028 ± 32	0.1 ± 0.0	81.1 ± 1.5	17.8 ± 1.5	15.8 ± 0.5
Bitter Nut Cracker with Chili	2014 ± 2	9.0 ± 1.4	72.9 ± 1.3	17.1 ± 0.1	15.1 ± 2.4
Mini Love Letter	2011 ± 12	22.3 ± 0.1	58.6 ± 0.5	17.4 ± 0.2	16.3 ± 0.2
Chocolate Wafer	1972 ± 9	22.3 ± 0.3	62.8 ± 0.1	15.1 ± 0.3	13.9 ± 0.0
Bitter Nut Crackers	1969 ± 5	10.6 ± 1.2	43.0 ± 0.4	28.4 ± 0.7	26.8 ± 0.1
Strawberry Wafer	1938 ± 2	18.4 ± 0.3	67.1 ± 0.4	13.5 ± 0.2	12.0 ± 0.1
Nyonya Pandan Egg Roll	1935 ± 11	16.6 ± 0.8	69.2 ± 1.3	13.3 ± 0.5	14.6 ± 0.2
Peanut Cookies	1854 ± 9	33.5 ± 0.6	56.0 ± 1.0	9.5 ± 0.4	22.2 ± 0.1
Kueh Bahulu	1796 ± 18	27.3 ± 0.1	62.7 ± 1.1	7.7 ± 0.1	4.3 ± 0.0
Sunflower Pineapple Tart	1785 ± 86	0.1 ± 0.0	85.3 ± 7.0	9.4 ± 0.8	7.1 ± 0.2
Nyonya Pineapple Roll	1652 ± 60	0.1 ± 0.0	75.4 ± 3.6	10.2 ± 0.6	7.4 ± 0.3
Cake Bangkit	1587 ± 17	21.7 ± 0.1	51.2 ± 0.7	9.7 ± 0.2	7.1 ± 0.0

**Table 2:** Fatty acid profiles (% of total fatty acids) * of the commonly consumed CNY snacks.

Snack Item	C8:0	C10:0	C12:0	C14:0	C16:0	C18:0	C22:0	C24:0	C16:1	C18:1	C20:1	C18:2	C18:3
Salted Egg	-	-	-	1.80 ± 0.01	32.93 ± 0.00	8.06 ± 0.04	-	-	1.09 ± 0.01	38.10 ± 0.12	-	14.63 ± 0.04	-
Fish Skin													
Arrowhead	-	-	-	1.65 ± 0.02	37.66 ± 0.05	7.33 ± 0.15	-	-	-	39.76 ± 0.00	-	11.66 ± 0.18	-
Cracker													
Peanut Puff	-	-	-	1.03 ± 0.01	29.49 ± 0.02	6.22 ± 0.02	2.02 ± 0.02	-	-	36.82 ± 0.13	-	21.68 ± 0.07	-
Egg Crisp	-	-	-	1.30 ± 0.00	32.10 ± 0.09	7.83 ±0.02	-	-	-	38.24 ± 0.16	-	18.13 ± 0.26	-
Plait Cookies	-	-	1.49 ± 0.04	1.90 ± 0.08	35.84 ± 0.23	7.34 ± 0.21	-	-	-	38.11 ± 0.28	-	13.82 ± 0.10	-
Peanut	-	-	-	-	15.94 ± 0.04	5.69 ± 0.05	5.08 ± 0.04	2.38 ± 0.05	-	35.98 ± 0.10	1.57 ± 0.02	32.77 ± 0.04	-
Candy													
Cashew Nut	1.25 ± 0.03	1.89 ± 0.04	9.89 ± 0.03	7.92 ± 0.08	27.37 ± 0.20	10.43 ± 0.05	-	-	-	28.06 ± 0.08	-	9.19 ± 0.03	-
Cookies													
Seaweed	-	-	-	1.66 ± 0.04	34.86 ± 0.21	7.03 ± 0.14	-	-	-	40.21 ± 0.11	-	14.16 ± 0.06	-
Roll													
Prawn Roll	-	-	3.06 ± 0.01	2.35 ± 0.04	27.93 ± 0.09	6.00 ± 0.16	-	-	-	36.81 ± 0.09	-	16.51 ± 0.09	5.01 ± 0.00
Green Pea	-	-	-	1.37 ± 0.00	35.83 ± 0.02	6.70 ± 0.01	-	-	-	39.67 ± 0.11	-	14.61 ± 0.08	-
Cookies													
Peanut	-	-	-	-	24.63 ± 0.07	6.44 ± 0.12	2.27 ± 0.08	1.05 ± 0.06	-	37.33 ± 0.24	-	25.81 ± 0.04	-
Sesame													
Almond	-	-	-	1.36 ± 0.06	33.49 ± 0.13	6.59 ± 0.21	-	-	-	41.87 ± 0.13	-	15.05 ± 0.05	-
Cookies													
Almond	1.17 ± 0.01	2.19 ± 0.01	11.20 ± 0.04	10.45 ± 0.01	20.05 ± 0.02	13.20 ± 0.01	-	-	1.29 ± 0.00	27.58 ± 0.01	-	8.64 ± 0.11	-
Cashew													
Chocolate													
Cookies													
Vegetarian	-	-	-	1.31 ± 0.02	41.80 ± 0.49	6.60 ± 0.07	-	-	-	36.67 ± 0.19	-	12.59 ± 0.58	-
Almond													
cookies													
Oat and Bran	-	-	8.20 ± 0.04	4.73 ± 0.03	27.75 ± 0.04	8.91 ± 0.04	-	-	-	32.22 ± 0.01	-	14.36 ± 0.02	-
Cookies													
Salted Egg	-	-	3.60 ± 0.04	2.60 ± 0.03	35.85 ± 0.24	7.12 ± 0.04	-	-	-	36.45 ± 0.04	-	12.38 ± 0.38	-
Yolk													
Cookies													
Almond	-	-	1.26 ± 0.04	3.15 ± 0.03	16.05 ± 0.05	5.41 ± 0.06	-	-	-	42.24 ± 0.14	-	27.69 ± 0.09	-
Delight													
Butter	1.44 ± 0.02	2.49 ± 0.03	10.44 ± 0.00	10.13 ± 0.03	27.96 ± 0.09	10.93 ± 0.04	-	-	1.11 ± 0.03	24.15 ± 0.13	-	6.06 ± 0.06	-
Cookies													
Sambal Chili	-	-	-	1.56 ± 0.03	36.90 ± 0.09	7.16 ± 0.20	-	-	-	39.50 ± 0.06	-	12.79 ± 0.13	-
Tapioca													
Crisp													
Bitter Nut	-	-	-	1.29 ± 0.00	34.80 ± 0.05	8.32 ± 0.06	-	-	-	40.51 ± 0.02	-	13.39 ± 0.01	-
Cracker with													
Chili													
Mini Love	7.57 ± 0.07	7.37 ± 0.08	30.59 ± 0.05	19.15 ± 0.07	13.49 ± 0.05	5.16 ± 0.07	-	-	-	11.59 ± 0.03	-	3.88 ± 0.27	-
letter													
Chocolate	-	-	-	1.56 ± 0.00	34.77 ± 0.03	8.31 ± 0.01	-	-	-	34.53 ± 0.06	-	17.37 ± 0.00	1.40 ± 0.02
wafer													
Bitter Nut	-	-	-	1.51 ± 0.01	36.00 ± 0.29	7.04 ± 0.06	-	-	-	40.17 ± 0.14	-	13.60 ± 0.32	-
Crackers													
Strawberry	-	-	-	1.79 ± 0.06	34.92 ± 0.31	7.50 ± 0.17	-	-	-	35.96 ± 0.24	-	16.31 ± 0.14	1.15 ± 0.08
Wafer													
Nyonya	-	-	-	1.78 ± 0.09	40.11 ± 0.16	8.10 ± 0.04	-	-	-	35.83 ± 0.20	-	12.11 ± 0.02	-
Pandan Egg													
Roll													
Peanut	-	-	-	-	16.06 ± 0.13	5.73 ± 0.04	5.26 ± 0.03	2.61 ± 0.00	-	36.78 ± 0.25	1.59 ± 0.02	31.56 ± 0.46	-
Cookies													
Kueh Bahulu	-	-	-	-	27.62 ± 0.01	6.96 ± 0.10	-	-	1.97 ± 0.03	38.43 ± 0.05	-	21.08 ± 0.09	1.18 ± 0.01
Sunflower	-	-	11.08 ± 0.00	5.65 ± 0.03	33.21 ± 0.07	6.41 ± 0.01	-	-	-	30.98 ± 0.09	-	9.58 ± 0.20	-
Pineapple													
Tart													
Nonya	-	-	8.89 ± 0.32	4.86 ± 0.22	32.92 ± 0.05	7.58 ± 0.09	-	-	-	32.22 ± 0.27	-	10.66 ± 0.48	-
Pineapple													
Roll													
Cake	6.20 ± 0.09	6.01 ± 0.12	24.51 ± 0.29	15.95 ± 0.23	15.53 ± 0.41	5.79 ± 0.00	-	-	-	17.77 ± 0.33	-	6.07 ± 0.05	-
Bangkit													

*: only fatty acids content more than 1% will be reported.

**Table 3:** Mineral contents of the commonly consumed CNY snacks. Values are expressed as mean ± SD.

Snack Item	Sodium (mg/100 g)	Magnesium (mg/100 g)	Aluminium (mg/100 g)	Potassium (mg/100 g)	Calcium (mg/100 g)	Manganese (mg/100 g)	Iron (mg/100 g)	Copper (mg/100 g)	Zinc (mg/100 g)
Salted Egg Fish Skin	551.8 ± 29.0	10.1 ± 0.1	0.1 ± 0.0	61.8 ± 0.5	382.3 ± 26.0	0.0 ± 0.0	1.9 ± 0.3	0.0 ± 0.0	1.4 ± 0.0
Arrowhead Cracker	36.3 ± 22.3	57.3 ± 27.4	0.1 ± 0.0	1300.6± 627.6	113.4 ± 54.4	0.4 ± 0.2	1.4 ± 0.7	0.1 ± 0.1	1.2 ± 0.8
Peanut Puff	77.2 ± 1.3	47.7 ± 0.9	0.4 ± 0.0	129.3 ± 1.7	146.5 ± 0.4	0.4 ± 0.0	2.0 ± 0.0	0.2 ± 0.0	1.2 ± 0.0
Egg Crisp	15.8 ± 0.0	26.8 ± 0.3	0.1 ± 0.0	69.6± 0.2	101.4 ± 1.7	0.2 ± 0.0	0.7 ± 0.0	0.1 ± 0.0	0.5 ± 0.0
Plait Cookies	225.5 ± 10.7	12.2 ± 0.6	0.1 ± 0.1	44.5± 1.4	45.1 ± 1.9	0.2 ± 0.0	0.5 ± 0.0	0.0 ± 0.0	0.3 ± 0.1
Peanut Candy	12.0 ± 0.5	83.8 ± 0.5	0.1 ± 0.0	204.1 ± 1.1	160.6 ± 0.4	0.5 ± 0.0	5.8 ± 5.5	0.2 ± 0.0	1.0 ± 0.0
Cashew Nut Cookies	146.0 ± 2.1	22.2 ± 0.3	0.1 ± 0.0	74.5 ± 48.4	95.8 ± 1.6	0.3 ± 0.0	0.7 ± 0.0	0.1 ± 0.0	0.5 ± 0.0
Seaweed Roll	413.4 ± 2.1	26.6 ± 0.5	0.6 ± 0.0	135.0 ± 5.7	183.9 ± 7.5	0.3 ± 0.0	1.4 ± 0.0	0.1 ± 0.0	0.3 ± 0.0
Prawn Roll	333.0 ± 4.3	46.2 ± 1.9	0.8 ± 0.0	93.4± 1.0	734.8 ± 32.9	0.3 ± 0.0	1.6 ± 0.4	0.3 ± 0.0	0.6 ± 0.0
Green Pea Cookies	171.6 ± 2.0	23.3 ± 0.5	0.3 ± 0.0	103.0 ± 0.9	92.2 ± 3.2	0.4 ± 0.0	0.9 ± 0.0	0.1 ± 0.0	0.5 ± 0.0
Peanut Sesame	34.3 ± 2.6	64.2 ± 6.5	0.2 ± 0.0	141.6 ± 18.5	225.4 ± 23.3	0.5 ± 0.0	1.4 ± 0.0	0.2 ± 0.0	0.8 ± 0.0
Almond Cookies	118.2 ± 5.8	18.3 ± 0.3	0.1 ± 0.0	63.1± 0.6	109.1 ± 4.2	0.3 ± 0.0	0.6 ± 0.2	0.1 ± 0.0	0.2 ± 0.0
Almond Cashew Chocolate Cookies	245.3 ± 2.0	43.6 ± 0.7	0.5 ± 0.0	167.0 ± 2.8	233.5 ± 6.2	0.4 ± 0.0	2.2 ± 0.1	0.3 ± 0.0	0.7 ± 0.0
Vegetarian Almond cookies	143.7 ± 2.7	22.7 ± 1.0	0.1 ± 0.0	62.6± 4.4	134.6 ± 10.9	0.2 ± 0.0	0.4 ± 0.1	0.1 ± 0.0	0.2 ± 0.0
Oat and Bran Cookies	185.7 ± 7.2	26.2 ± 0.1	0.1 ± 0.0	94.0± 1.0	138.3 ± 1.4	0.7 ± 0.0	0.8 ± 0.0	0.1 ± 0.0	0.4 ± 0.0
Salted Egg Yolk Cookies	267.3 ± 10.9	8.5 ± 0.0	0.3 ± 0.0	52.5± 0.3	120.0 ± 0.0	0.2 ± 0.0	1.2 ± 0.2	0.0 ± 0.0	0.3 ± 0.0
Almond Delight	268.2 ± 7.0	46.4 ± 0.1	27.8 ± 0.2	158.3 ± 0.6	409.1 ± 0.3	0.5 ± 0.0	1.7 ± 0.1	0.2 ± 0.0	0.6 ± 0.0
Butter Cookies	111.6 ± 0.4	11.1± 0.0	0.2 ± 0.2	45.6± 0.3	169.9 ± 1.4	0.2 ± 0.0	0.7 ± 0.1	0.0 ± 0.0	0.3 ± 0.0
Sambal Chili Tapioca Crisp	251.8 ± 14.2	16.5 ± 0.6	0.6 ± 0.6	169.2 ± 3.0	131.4 ± 3.8	0.1 ± 0.0	0.8 ± 0.1	0.0 ± 0.0	0.3 ± 0.1
Bitter Nut Cracker with Chili	98.1 ± 0.3	25.6 ± 0.1	0.6 ± 0.6	132.1 ± 1.1	102.1 ± 0.5	1.3 ± 0.0	1.2 ± 0.2	0.1 ± 0.0	0.5 ± 0.1
Mini Love letter	10.5 ± 0.0	27.3 ± 0.4	0.2 ± 0.0	141.2 ± 0.9	88.2 ± 0.9	0.5 ± 0.0	4.7 ± 0.0	0.1 ± 0.0	2.1 ± 0.0
Chocolate wafer	16.5 ± 0.3	24.3 ± 0.6	0.7 ± 0.0	123.6 ± 5.7	184.8 ± 7.8	0.3 ± 0.0	2.3 ± 0.2	0.1 ± 0.0	0.4 ± 0.0
Bitter Nut Crackers	0.4 ± 0.0	40.2 ± 1.0	1.2 ± 0.1	271.7 ± 1.2	168.8 ± 0.1	2.2 ± 0.1	1.9 ± 0.0	0.3 ± 0.0	0.8 ± 0.0
Strawberry Wafer	5.3 ± 0.1	11.9 ± 0.0	0.4 ± 0.0	62.9 ± 0.5	126.7 ± 1.2	0.2 ± 0.0	1.0 ± 0.1	0.0 ± 0.0	0.2 ± 0.0
Nyonya Pandan Egg Roll	137.1 ± 2.4	16.2 ± 0.8	0.1 ± 0.0	104.0 ± 1.7	344.6 ± 12.6	0.3 ± 0.0	0.5 ± 0.0	0.0 ± 0.0	0.2 ± 0.0
Peanut Cookies	3.0 ± 0.0	56.6 ± 0.1	0.1 ± 0.0	152.8 ± 2.1	109.1 ± 2.8	0.5 ± 0.0	0.9 ± 0.1	0.1 ± 0.0	1.0 ± 0.4
Kueh Bahulu	98.5 ± 7.0	10.5 ± 0.6	0.1 ± 0.0	72.0 ± 5.1	117.7 ± 6.1	0.2 ± 0.0	1.0 ± 0.0	0.0 ± 0.0	0.3 ± 0.0
Sunflower Pineapple Tart	123.3 ± 0.5	10.4 ± 0.2	0.0 ± 0.0	63.8 ± 0.1	76.6 ± 1.1	0.7 ± 0.0	0.5 ± 0.1	0.0 ± 0.0	0.1 ± 0.1
Nonya Pineapple Roll	152.6 ± 4.3	10.5 ± 0.4	0.0 ± 0.0	66.8 ± 2.2	67.8 ± 0.5	0.6 ± 0.0	0.5 ± 0.3	0.0 ± 0.0	0.1 ± 0.1
Cake Bangkit	11.5 ± 0.2	8.2 ± 0.2	0.5 ± 0.0	47.5 ± 1.0	163.1 ± 2.1	0.2 ± 0.0	0.6 ± 0.0	0.0 ± 0.0	0.2 ± 0.0

**Table 4:** Nutrient contents of 50 g selected snacks. Percent Daily Value based on an 8368 kJ (2000 kCal) diet.

Amount	% Daily Value	Amount	% Daily Value	Amount	% Daily Value	Amount	% Daily Value	Amount	% Daily Value
Salted Egg Fish Skin		Peanut Candy		Prawn Roll		Mini Love Letter		Sunflower Pineapple Tart	
Energy 1372 kJ	16%	Energy 1166 kJ	14%	Energy 1124 kJ	13%	Energy 1006 kJ	12%	Energy 893 kJ	11%
Total CHO 2 g	1%	Total CHO 24 g	8%	Total CHO 25 g	8%	Total CHO 29 g	10%	Total CHO 43 g	14%
Total Fat 26 g	40%	Total Fat 16 g	25%	Total Fat 14 g	22%	Total Fat 9 g	14%	Total Fat 5 g	8%
Sat Fat 12 g	60%	Sat Fat 5 g	25%	Sat Fat 6 g	30%	Sat Fat 8 g	67%	Sat Fat 3 g	25%
Na 276 mg	11%	Na 6 mg	0.3%	Na 167 mg	7%	Na 5 mg	0.2%	Na 62 mg	3%
Ca 191 mg	19%	Ca 80 mg	8%	Ca 367 mg	37%	Ca 44 mg	4%	Ca 38 mg	4%
Fe 1.0 mg	12%	Fe 2.9 mg	36%	Fe 0.8 mg	10%	Fe 2.4 mg	30%	Fe 0.2 mg	3%
Zn 0.7 mg	7%	Zn 0.5 mg	5%	Zn 0.3 mg	3%	Zn 1.0 mg	10%	Zn 0.05 mg	0.5%

## Supplementary Materials

The following are available online at https://www.mdpi.com/2304-8158/9/8/1137/s1, Figure S1: 50 g portion size of the Chinese New Year snacks, Table S1: Spike recovery test (Arrow Head Cracker), Table S2: Spike recovery test (Salted Egg Fish Skin), Table S3: Spike recovery test (Kueh Bangkit).
