# Supplementary material for: Energy Density and Nutrient Contents of Selective Chinese New Year Snacks
Source: Foods. 2020 Aug 18;9(8):1137. doi: 10.3390/foods9081137 (PMC7466284; doi:10.3390/foods9081137)
Supplement: Supplementary file 1 [file foods-09-01137-s001.pdf]

	Unit	Spiked Amount	Sample Blank	Recovery	Recovery (%)
Na	mg/L	50	2.12	53.99	103.72
Mg	mg/L	5	3.93	9.35	108.23
Al	mg/L	5	0.00	5.47	109.39
K	mg/L	50	10.08	62.21	104.26
Ca	mg/L	50	4.37	56.87	105.01
Mn	mg/L	0.05	0.03	0.08	109.23
Fe	mg/L	5	0.08	5.48	107.96
Cu	mg/L	0.05	0.01	0.06	106.17
Zn	mg/L	5	0.07	5.16	101.67

Table S1: Spike recovery test (Arrow Head Cracker).

	Unit	Spiked Amount	Sample Blank	Recovery	Recovery (%)
Na	mg/L	50	2.12	53.99	103.72
Mg	mg/L	5	1.01	6.42	108.27
Al	mg/L	5	0.01	5.36	107.11
K	mg/L	50	6.19	11.67	109.51
Ca	mg/L	50	3.74	52.74	98.00
Mn	mg/L	0.05	0.00	0.06	104.04
Fe	mg/L	5	0.15	5.45	106.13
Cu	mg/L	0.05	0.00	0.06	102.54
Zn	mg/L	5	0.15	5.45	106.13

Table S2: Spike recovery test (Salted Egg Fish Skin).

	Unit	Spiked Amount	Sample Blank	Recovery	Recovery (%)
Na	mg/L	50	2.15	55.09	105.89
Mg	mg/L	5	1.73	7.15	108.40
Al	mg/L	5	0.10	5.55	109.02
K	mg/L	50	10.19	63.49	106.59
Ca	mg/L	50	4.42	58.04	107.24
Mn	mg/L	0.05	0.03	0.09	108.11
Fe	mg/L	5	0.09	5.43	106.73
Cu	mg/L	0.05	0.01	0.06	109.97
Zn	mg/L	5	0.04	5.12	101.52

Table S3: Spike recovery test (Kueh Bangkit).

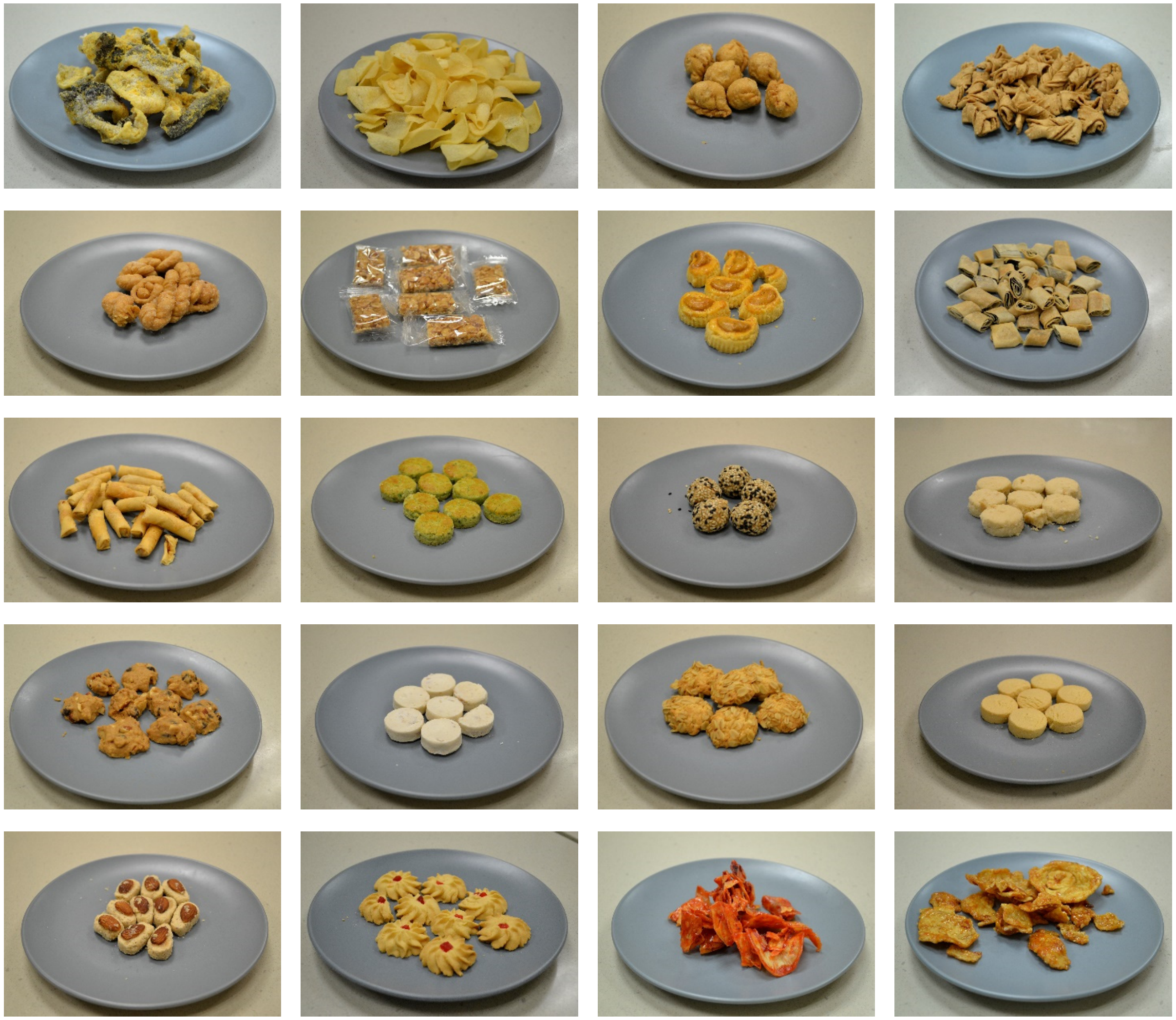

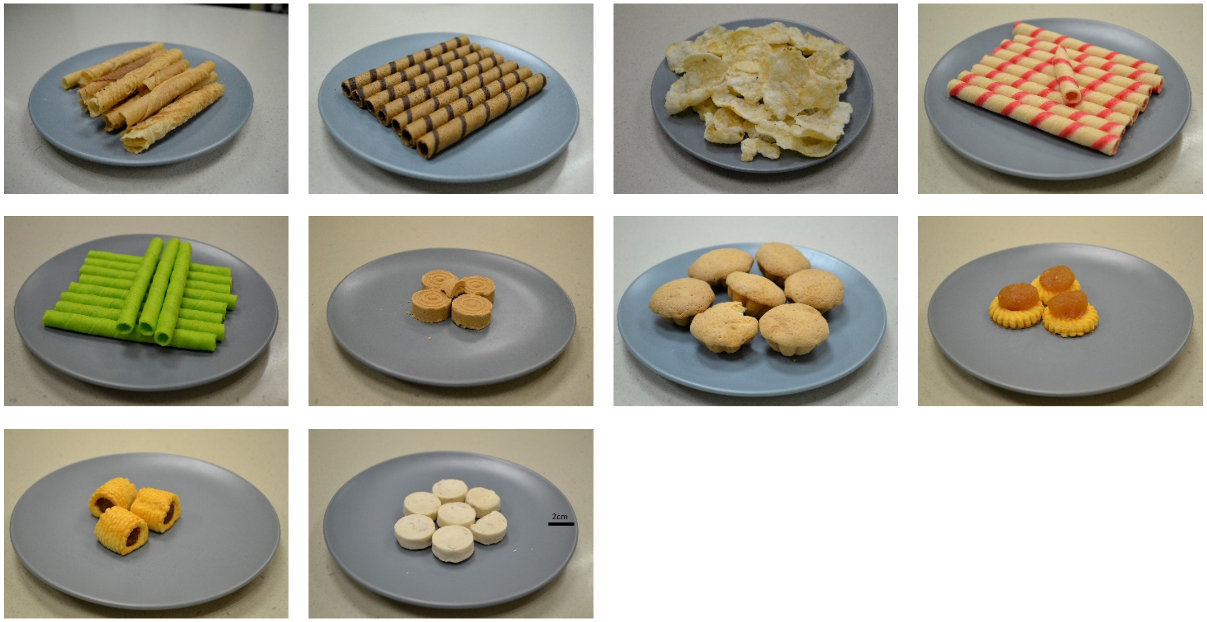

Figure S1: 50g portion size of the Chinese New Year snacks.
Row 1 (from left to right): Salted Egg Fish Skin, Arrowhead Cracker, Peanut Puff, Egg Crisp.
Row 2 (from left to right): Plait Cookies, Peanut Candy, Cashew Nut Cookies, Seaweed Roll
Row 3 (from left to right): Prawn Roll, Green Pea Cookies, Peanut Sesame, Almond Cookies
Row 4 (from left to right): Almond Cashew Chocolate Cookies, Vegetarian Almond Cookies, Oat and Bran Cookies, Salted Egg Yolk cookies
Row 5 (from left to right): Almond Delight, Butter Cookies, Sambal Chili Tapioca Crisp, Bitter Nut Cracker with Chili
Row 6 (from left to right): Mini Love Letter, Chocolate Wafer, Bitter Nut Crackers, Strawberry Wafer
Row 7 (from left to right): Nyonya Pandan Egg Roll, Peanut Cookies, Kueh Bahulu, Sunflower Pineapple Tart
Row 8: (from left to right): Nyona Pineapple Roll, Cake Bangkit.
Scale bar: 2cm
